# Distance-based phenotypic association analysis of DNA sequence data

**DOI:** 10.1186/1753-6561-5-S9-S54

**Published:** 2011-11-29

**Authors:** Doyoung Chung, Qunyuan Zhang, Aldi T Kraja, Ingrid B Borecki, Michael A Province

**Affiliations:** 1 Division of Statistical Genomics, Department of Genetics, Washington University School of Medicine, St. Louis, MO 63110, USA

## Abstract

As the cost of sequencing decreases, the demand for association tests that use exhaustive DNA sequence information increases. One such association test is multivariate distance matrix regression (MDMR). We explore some of the features of MDMR using Genetic Analysis Workshop 17 simulated data in search of potential improvements in distance measures. We used genotype data from 697 unrelated individuals, in 200 replications, to test the power of MDMR to detect 13 trait Q2 causative genes based on the Euclidean distance metric. We also estimated the false-positive rate of MDMR using 508 control genes. In addition, we compared MDMR with Mantel’s test and collapsing analysis for rare variants. MDMR performed comparably well even with the Euclidean distance measure.

## Background

High-throughput sequencing technology allows identification of new rare alleles in a human population, but the sparseness of these alleles in samples becomes an important obstacle to detecting the true effects of rare variants under a single-variant-based paradigm. The increasing number of identified genetic variants also requires more statistical tests, thereby aggravating the issue of low statistical power. Several methods, based on collapsing or grouping rare variants, have been developed to alleviate this problem. However, these methods arbitrarily define common and rare variants and treat them differently for analysis. This artificial classification may fail to capture important biological reality. For example, it has been shown that a common variant can act as a modifier of a rare variant’s effect [1,2]. It is quite possible that collapsing multiple rare variants may dilute the true genetic effect.

Multivariate distance matrix regression (MDMR) can provide a flexible platform for a phenotypic association test of DNA sequence [3]. In this method, for a region of DNA sequence, genotype dissimilarities between individuals are associated with phenotype dissimilarities. MDMR does not test a single variant but instead aggregates each genetic variant’s information to build a distance matrix. For this reason, once an appropriate distance matrix is assumed, the issues of multiple testing and sparseness of data become less critical for MDMR. The most critical problem in MDMR is how to define a distance metric for summarizing genetic differences between individuals in relation to their influence on trait(s) or disease(s). Different distance measures can be used; one of them is the Euclidean distance. Similarity-based association tests such as MDMR can be as powerful as some traditional tests of association when common variations are involved [4]. However, the utility of these methods for rare variant analysis is not well understood and needs to be interrogated [5].

## Methods

### Multivariate distance matrix regression

MDMR is based on the multivariate multiple regression model [3,6,7] defined in matrix notation as:(1)

where **Y** is an *n* × *p* data matrix, in which *n* is the number of subjects and *p* is the number of unknown biologically relevant genetic variables; and **X** is an *n* × *m* model matrix, in which *m* is the number of predictor variables. *β* is an *m* × *p matrix of beta coefficients and ε* is an *n* × *p* residual matrix. Because genotype is regressed using a combination of traits and covariates under this model, *m* regressors can be phenotypes (e.g., Q1, Q2, and Q4 in the Genetic Analysis Workshop 17 [GAW17] data) and covariates (e.g., Sex, Age, and Smoke in the GAW17 data). A pseudo-*F* statistic can be constructed to test the null hypothesis of *β* = 0:(2)

where the projection matrix is:(3)

and the **G** matrix is Gower’s centered matrix, which can be calculated from an *n* × *n* distance matrix **D**. The **G** matrix replaces **YY′** and can be calculated from any symmetric distance matrix allowing for nonmetric dissimilarity measures. The **I** matrix is the *n* × *n* identity matrix, and tr stands for the trace of matrix. To assess statistical significance of the pseudo-*F* statistic, one can use permutation tests.

### MDMR as a gene-based association test

We calculated Euclidean distances using numerically coded genotypes of 13 Q2 risk genes for all possible pairs of the 697 unrelated individuals:(4)

where the Euclidean distance is defined as the L2 norm between two individual genotype vectors **a** and **b**. Genotypes were coded as the number of minor alleles with no weighting of single-nucleotide polymorphisms (SNPs) was applied. For each gene and each Q2 simulation, we constructed a 697 × 697 genotypic distance matrix **D** and a 697 × 1 phenotype matrix **X**, which consists of the individual Q2 trait values, and used them to calculate a pseudo-*F* statistic under the regression model that includes the Q2 trait as the sole independent variable. Each of the 13 × 200 tests underwent 1,000 permutations in which the rows and columns of its raw genotype matrix (i.e., the individual-by-SNP matrix) were shuffled at random. The empirical *p*-value was determined as the frequency of observing more extreme pseudo-*F* statistics in permutations than in the actual gene case. MDMRs were performed either using all variants within a gene or using only rare variants with minor allele frequency (MAF) less than 0.01. Similarly, we selected 508 noncausative (i.e., control) genes for Q2 and tested them using all 200 replications. We omitted a subset containing 125 genes from these 508 control genes for the rare-variant-only analyses because they contained no rare variants.

### Mantel test

The Mantel test measures the correlation between two distance matrices [8]. In our application, we calculated a phenotypic distance matrix and a genotypic distance matrix based on the Euclidean distance measure. The two distance matrices were then tested for correlation [9]. The genotypic distance matrix for the Mantel test was identical with that of the MDMR, whereas a 697 × 697 distance matrix was calculated for each Q2 simulated replicate. Mantel tests were performed for the 13 Q2 risk genes using either all variants or only rare variants. Similarly, 508 control genes were tested for association using all variants, among which 383 genes continued to be tested using only rare variants. *P-values* were empirically determined using 1,000 permutations. We estimated the power and false-positive rates on the basis of the significance threshold value of 0.05 and compared them with the values from MDMR and collapsing analysis.

### Collapsing analysis

Collapsing analysis is a simple regression analysis that uses a collapsed variable [10] into which rare variants are collapsed in a binary manner based on the presence of any rare variant. Because our collapsing analysis excluded all “common” variants (defined by MAF > 0.01), we also removed common variants in the other analyses to facilitate comparison. This allowed 12 Q2 risk genes to be compared, because one risk gene had no rare variants. Similarly, we tested 380 selected genes, simulated under the null hypothesis for Q2, for association with Q2 using all three methods. No correction for population structure or hidden relatedness was applied throughout this study.

## Results

Table 1 shows the statistical powers of five different strategies for the 13 Q2 risk genes: MDMR using all variants, Mantel test using all variants, MDMR using only rare variants, Mantel test using only rare variants, and collapsing analysis using only rare variants. The estimated power varied extensively depending on the gene simulation and the method used. For example, *VNN1* was significantly (*p* < 0.05) associated with Q2 in 94% of the total replicates when MDMR using all variants was used. The Mantel test, however, discovered association in only 25% of *VNN1* replicates and failed to find any risk gene with a detection rate greater than 50% regardless of the MAF-based SNP filtering.

**Table 1:** True positive rates of five different strategies for the 13 Q2 risk genes

Gene	Setting	MDMR using all variants	Mantel test using all variants	MDMR using only rare variants	Mantel test using only rare variants	Collapsing analysis using only rare variants
*BCHE*	1c + 28r (13s)	0.045	0.170	0.320	0.310	0.455
*GCKR*	1c (1s)	0.405	0.150	NA	NA	NA
*INSIG1*	1c + 4r (3s)	0.090	0.020	0.040	0.040	0.035
*LPL*	5c (1s) + 15r (2s)	0.045	0.135	0.060	0.125	0.040
*PDGFD*	5c + 6r (4s)	0.065	0.035	0.685	0.290	0.745
*PLAT*	4c + 25r (8s)	0.035	0.030	0.055	0.040	0.110
*RARB*	2c + 9r (2s)	0.105	0.145	0.410	0.115	0.155
*SIRT1*	1c + 23r (9s)	0.365	0.285	0.605	0.320	0.330
*SREBF1*	3c + 21r (10s)	0.030	0.110	0.380	0.205	0.690
*VLDLR*	4c + 23r (8s)	0.055	0.065	0.140	0.140	0.140
*VNN1*	1c (1s) + 6r (1s)	0.940	0.250	0.200	0.085	0.050
*VNN3*	6c (3s) + 9r (4s)	0.190	0.175	0.025	0.055	0.030
*VWF*	2c + 6r (2s)	0.180	0.080	0.285	0.080	0.190
Mean		0.196	0.127	0.267	0.150	0.248

The true positive rate was determined as the frequency of observing *p*-values less than 0.05 among 200 (replication) *p*-values for each gene. The “Setting” column shows the composition of SNPs within a gene: c, r, and s stand for common, rare, and signal SNPs, respectively. For example, *VNN1* has 1 common causal SNP and 6 rare SNPs, one of which is a signal. SNPs with MAF > 0.01 are defined as common.

When variants with MAF > 0.01 were removed, MDMR identified two genes, *PDGFD* and *SIRT1*, with power greater than 50%. *VNN1* did not survive the cutoff this time, presumably because one of its causal SNPs was common and thus removed from the analysis. The common variants of *PDGFD* and *SIRT1* were all noncausal, and removal of these variants may have enhanced performance of MDMR on these genes. *PDGFD* was also found by collapsing analysis using the cutoff value of 50%, along with *SREBF1*. The estimated power for the 12 Q2 risk genes was comparable between the Euclidean MDMR and collapsing analysis, whereas the Mantel test appeared to be less sensitive than the other methods.

**Figure 1:**
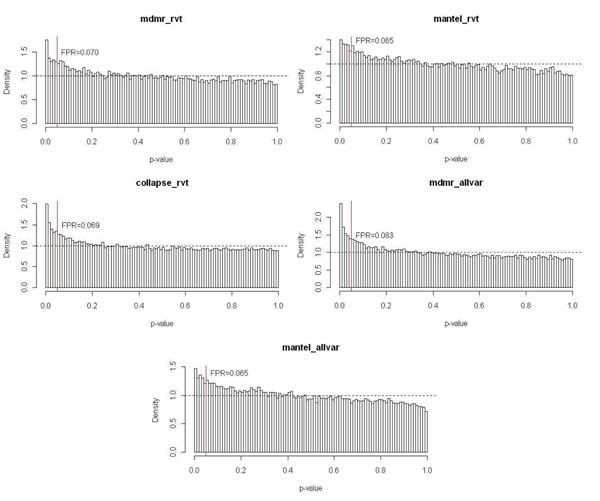
**False positive rates of five strategies.** mdmr_rvt, MDMR using only rare variants; mantel_rvt, Mantel test using only rare variants; collapse_rvt, collapsing analysis using only rare variants; mdmr_allvar, MDMR using all variants; mantel_allvar, Mantel test using all variants. The red vertical lines mark the significance threshold *p*-value of 0.05.

Because MDMR is computationally intensive, we focused on only 508 control genes to compare the false-positive rates of all three methods. The false-positive rates of MDMR and collapsing analysis were similar and slightly inflated (Figure 1). The Mantel test produced a less inflated type I error rate, suggesting that this method may be more conservative than the other methods. This type I error inflation can be primarily attributed to the lack of correction for population stratification or any hidden relatedness. However, it is unclear whether population structure alone can explain the inflation.

**Figure 2:**
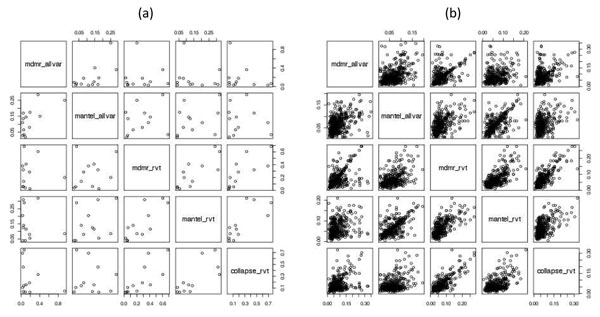
**Pairwise scatterplots between five different strategies**. (a) Estimated power of detecting the Q2 risk genes for a pair of methods. Each point represents a Q2 risk gene whose coordinate indicates the power estimates from two different strategies. (b) False-positive rate of Q2 control genes for a pair of methods. Each point represents a Q2 control gene whose coordinate indicates the false-positive rates from a pair of strategies. mdmr_rvt, MDMR using only rare variants; mantel_rvt, Mantel test using only rare variants; collapse_rvt, collapsing analysis using only rare variants; mdmr_allvar, MDMR using all variants; mantel_allvar, Mantel test using all variants.

Although the best performing method differed from gene to gene, the power and false-positive rate of MDMR, the Mantel test, and collapsing analysis were inclined to be positively correlated, implying that there is a general agreement in performance between these methods (Figure 2). Causal genes detected by one method tended to be detected by another method, and false-positive genes found in one method tended to be falsely detected in another method. In the presence of causal variants, however, the correlation could disappear or even become negative (Figure 2a). This phenomenon occurred when different sets of single-nucleotide polymorphisms (SNPs) were analyzed, that is, all variants vs. rare variants only. For example, Spearman’s correlation coefficient was 0.049 between MDMR using all variants and MDMR using only rare variants and −0.277 between MDMR using all variants and collapsing analysis using only rare variants. Therefore SNP selection can be critical when we test causal genes because we want to include causal variants and exclude noncausal variants for our analysis.

## Discussion

MDMR is a statistical method to test the aggregate effect of genetic variants based on a pairwise genotypic distance matrix. We applied the Euclidean MDMR to the GAW17 simulated data to compute the power of the method for detecting causative Q2 genes. We estimated the power and false-positive rate of MDMR using 200 simulated replicates of the Q2 trait for 13 Q2 causative genes and 508 control genes, respectively. MDMR was compared with collapsing analysis and the Mantel test for its performance on rare variants. We observed that the Euclidean MDMR performs comparably to collapsing analysis. The Mantel test, another distance-based method, seemed to behave more conservatively than the other methods. Our study also suggests that MDMR will perform better than the Mantel test and collapsing analysis in some genetic settings, presumably depending on the number of causal and noncausal variants under interrogation, their respective effect sizes, and any dependencies among them. To dissect the effect of each individual factor on performance, we need to examine various rare variant analysis methods in more contrived, simulated settings that confine confounding variables.

Originally designed for a region of genome, MDMR anticipates multiple causal SNPs with joint actions on the phenotype(s). Thus the unit of test can be easily reduced to a functional domain or a small set of adjacent SNPs [5] if we expect multiple causal variants in that unit. Our results imply that variant selection can affect the performance of MDMR. Along with determining biologically relevant variants to analyze, calculating distances using selected variants is crucial to any distance-based method. The performance of MDMR would be improved significantly if one could invent a measure of genetic distance that closely reflects phenotypic dissimilarity. MDMR may be inappropriate for large studies because it is computationally intensive. This is the reason we sampled a subset of control genes to estimate false-positive rates. Our MDMR program was implemented in the R programming language. Thus the analysis could be expedited by using a faster language, such as C or Java.

## Conclusions

The Euclidean MDMR performed comparably to collapsing analysis to detect the Q2 causal genes. The Mantel test was less sensitive than these methods with a slightly reduced type I error rate. Potential progress can be made because the distance matrix appreciates genotypic dissimilarities relevant only to phenotypic dissimilarities.

## Competing interests

The authors declare that there are no competing interests.

## Authors’ contributions

DC conceived of the study, performed the statistical analysis and drafted the manuscript. QZ developed the analytical pipeline for collapsing analysis. ATK, IBB and MAP contributed ideas to improve the design and the manuscript. All authors read and approved the final manuscript.
